# Dartanan: Prototype evaluations of a serious game to engage children in the calibration of their hearing aid functionalities

**DOI:** 10.1177/20556683211021527

**Published:** 2021-07-07

**Authors:** Madeline Hallewell, Davide Salanitri, Mirabelle D’Cruz, Sue Cobb, Lorenzo Picinali, Emily Frost, Stefano Tamascelli, Harshada Patel, On behalf of the 3D Tune-In project consortium

**Affiliations:** 1 Human Factors Research Group, Faculty of Engineering University of Nottingham, Nottingham, UK; 2 Dyson School of Design Engineering, Faculty of Engineering, Imperial College London, London, UK; 3 XTeam Software Solutions SRLS, Rovigo, Italy

**Keywords:** Evaluation, hearing, human factors, self care, design requirements

## Abstract

**Introduction:**

It is notoriously difficult to obtain a perfect fitting of hearing aids (HAs) for children as they often struggle to understand their hearing loss well enough to discuss the fitting adequately with their audiologist. Dartanan is an ‘edutainment’ game developed to help children understand the functions of their HA in different sound contexts. Dartanan also has elements of a leisure game for all children, in order to create an inclusive activity.

**Methods:**

Game prototypes were evaluated during two formative evaluations and a summative evaluation. In total 106 children with and without hearing loss in Italy, Spain and the UK played Dartanan. A built-in virtual HA enabled children with hearing loss to use headphones to play.

**Results and conclusions:** During the formative stages, feedback was discussed during focus groups on factors such as the audiological aspects, the extent to which children learned about HA functions, accessibility and usability, and this feedback was presented to the developers. After redevelopment, a summative evaluation was performed using an online survey. It was concluded that the game had met the goals of helping children understand their HA functionalities and providing an inclusive activity. User-evaluations were crucial in the development of the app into a useful and useable service.

## Introduction

According to the World Health Organisation (WHO), there are 32 million children (under 15 years) with hearing loss worldwide.^1^ Hearing loss has many consequences for children. It is well documented that children with hearing loss are more likely to underachieve at school and have poorer language skills. A 2013 literature review revealed a number of studies that highlight poorer educational outcomes of those with hearing loss compared to those without.^2^ Children with hearing loss are more likely to have problems with their social and emotional development. Young children with hearing loss are reported to have fewer communication interactions, lower success rates when initiating conversations compared to peers without hearing loss and are less capable of maintaining engagement in interactions with peers.^3^ Moreover, those with hearing aids (HAs) (and cochlear implants) are typically judged to have poorer intelligibility of speech than those without hearing loss.^4^

One of the most effective ways in which children with hearing loss can be supported to develop effective communication practices is by being fitted with a HA. In a 2015 study, children with mild hearing loss who wore HAs full-time scored higher on language and speech perception measures than did those with hearing loss who did not use HAs or used them infrequently.^5^ Similar findings have been noted elsewhere.^6,7^ However, there is a segment of the population of children with hearing loss (most likely those with mild hearing loss) who discontinue or reduce their usage of HAs, usually during the pre-teen years which are a crucial time in their life for social, emotional and academic development.^8,9^ It is thought that these children would prefer to make do with their limited hearing than wear HAs as they are uncomfortable and are perceived as a social stigma.^10^

Two major factors that might be associated with the decline of usage are the perceived limited benefits of HAs in social settings^11^ and/or fatigue.^12^ Current practice in fitting children with HAs involves an audiologist selecting a HA and features most suited to the child’s hearing profile; the child then undergoes a trial period in which they use the HA, and then they revisit the audiologist to report any problems. The audiologist can then adjust the HA to attempt to solve these problems.^13^ Within this standard practice, it is difficult to perfectly match the HA fitting to the child’s hearing loss prescriptive targets to address the child’s unique hearing loss. Despite guidelines on the acceptable level of error in fittings of HAs (5 dB error), it has been observed that 60% of children in a cohort of 195 children had at least one HA that exceeded this level of error and 55% had two HAs exceeding the level of error.^14^ This mis-match between prescription and fitting values is due to a number of factors including the inadequate sensitivity of audiograms especially relating to high frequencies,^15^ a lack of, or inadequate, systematic verification,^16^ degree of hearing loss^14^ and even simply the child being uncooperative or unable to communicate effectively at their appointment. Possibly due to these fitting issues, another study found that 65% of a sample of 38 children with HAs stated that they would like to hear better *‘in the classroom, during conversations with family members, and when there was a need to hear from a distance’* (p.847).^17^ Thus, children who are struggling with feelings of social stigma are likely to decide that HAs are not worth the trouble if they do not actually improve their hearing and cause fatigue. Consequently, the post-fitting trial and validation period is crucial to establish the efficacy of the fitting and identify any problems with the HA that the child experiences in real life settings.

The validation process of a HA fitting is also considered to be a difficult task due to its subjective nature: patients need to give feedback about how well their HA works in different real-life scenarios in helping them to hear speech. For this reason, Mendel^18^ tested subjective and objective measures of hearing ability and concluded that both are needed in the successful evaluation of HA fittings. However, Dahl and Hanssen^19^ argue that there is a terminology difference between audiologists and their patients who have difficulties objectively describing their problems in terms that are useful to audiologists, and in some cases they may not know which problems are relevant to discuss during their audiological rehabilitation. This problem is likely to be intensified in children, who are typically less likely to be able to adequately describe (or remember) their problems in different situations. Moreover, children with hearing loss are thought to be less likely than those without hearing loss to possess the skills and confidence associated with self-advocacy; that is being able to assert their problems and seek/demand solutions.^20^

Of course caregiver reports from observations made of their children outside of the clinic can be used to evaluate HA effectiveness. However, it is noted that caregiver observations might not be entirely reliable owing to differences in caregivers’ ability to closely observe their child (most caregivers have other competing responsibilities), and because there is great variation in techniques that audiology professionals use to extract this information from caregivers.^21^ Moreover, some children may be subject to changes in their hearing in between audiology appointments^22^ meaning that their HA becomes inadequate whilst still providing some level of hearing. It seems pertinent to examine methods of enabling audiologists to engage with children themselves in order to gain useful information about their hearing abilities, as well as empower children to more effectively discuss their problems (and preferences) for HA fittings (settings and available features) with their caregivers and audiologists.

### Serious games

The 3D-Games for TUNing and lEarnINg about HAs (3DTI) European project sought to create digital games for HA users.^23^ Stakeholders (researchers, developers, practitioners) involved in the development of Games for Health (G4H)-^24^ recognise the value of patients playing games to learn about their condition. G4H are effective because of the intrinsic motivation, broad accessibility, appeal, broad applicability, cost effectiveness, ease of everyday life fit and direct wellbeing support that come with game play.^25^ Caldwell et al.^26^ provide a review of the patient empowerment effects that might be possible through gamification: patients come to feel they have control over, and more knowledge of their condition. It can be argued that playing a game might be more effective than traditional techniques for learning, if only because it is more interesting and engaging to play a game than read an instruction manual. Thus a gamification approach was taken throughout the development of a suite of games to teach people with HAs about the functionalities of their HA.

**Figure 1.**
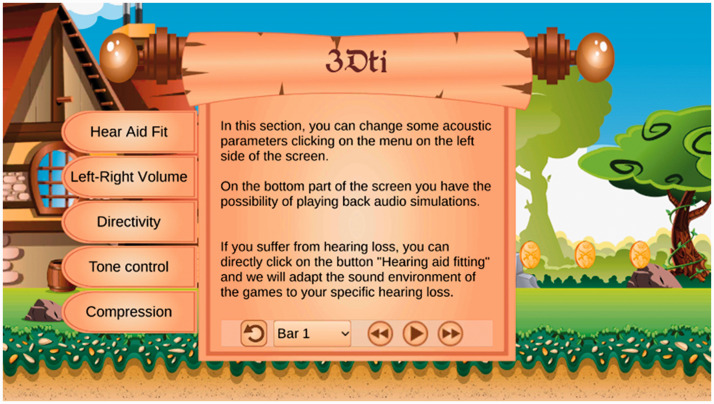
Screenshot of the introductory section for the 3DTI VHA for use by HA users.

These digital games make use of binaural (3D) sound technologies and a Virtual HA (VHA) toolkit (Figure 1),^27^ to perform the same functions as a real HA. The VHA enables HA users to experience sounds in realistic contexts and to adjust VHA settings to improve their hearing of stimulus sounds within those environments. HA users can test the different advanced settings and functions of HAs (e.g. compression, tone-control) in different scenarios (e.g. restaurants, busy streets). In order to progress through the levels of the game, HA users must be able to hear the stimulus sounds clearly, so they must calibrate the VHA to fit their level of hearing loss. In doing so, they learn about what the settings and functions can do to improve their hearing, and identify any enhancements that might be beneficial. The resulting data is fed back to the audiologist who can then use this information to perfect the user’s real HA fitting.

In recent years, several serious games have been developed to help people affected by disabilities. Examples are serious games for autism,^28,29^ cognitive impairments^30^ and physical disabilities.^31^ A promising area of research and development is the creation of disability-related simulations. An example is the work of Melthis and colleagues, aiming directly at visual impairment^32^ in which visual impairment was simulated for those without visual impairments in order to raise awareness. In terms of hearing impairment, several hearing loss simulators exist and are available as applications and/or web-based tools. These are mainly linked to hearing aid manufacturers, such as Starkey^I,II^ and Phonak,^III^ and to associations and charities.^IV,V^ These simulations are rather simplistic, and they are aimed at the general public, but uses of these applications for medical education and training has been successfully attempted.^33^ Serious games also have been developed for supporting patient treatment in the context of chronic hearing disorders, such as tinnitus,^34^ and a gamification approach was used to develop HA familiarisation games for adults with hearing loss.^35^ However, at the time of the 3DTI project no existing games for learning about the functionalities and effects of different HA settings were available for children.

The game Dartanan, developed by XTeam for the 3DTI project, helps children with hearing loss to learn about HA functions through engaging with a series of mini-games set within a main game which is aimed at all children. The game’s objective is to educate children on the usage of HAs, explaining the main functionalities and how to use them with the aim of helping children with hearing loss to become empowered to take control of their HAs, whilst also providing inclusive entertainment for all children. Children without hearing loss can play alongside their hearing-impaired peers, potentially increasing awareness of HA difficulties in this age group, whilst promoting empathy and understanding. The data gathered by the game from hearing impaired children can be processed to verify the settings of the HA and/or verify the state of the children’s hearing without HAs.

3DTI was an Innovation Action project, which partners academic institutions and companies, recognising that the development of apps outside of the developers usual/core business requires a different approach. One of the developers involved in this project, XTeam Software Solutions (XTeam), had not previously developed their products for the hearing loss community. Through being involved in this Innovation Action they aimed to gain a better understanding of this user-group in order to develop a game that would meet their specific needs.

This paper details the evaluation stages of the game’s development during which children with and without HAs were invited to evaluate working prototypes of the game for its utility (as a serious game) and playability (as an entertainment game).

## Materials and methods

### Dartanan

Dartanan is a traditional platform game and consists of a main game with levels that can be played by any child, and a series of mini-games designed specifically for children with HAs. It is an Android app that is designed for use on tablet devices. The game is presented with colourful two-dimensional (2D) graphics in order to appeal to younger users. It was considered that this style of game would be more familiar and easier to “pick-up-and-play” for children than a game with three-dimensional graphics with the more complicated controls that three-dimensional worlds would necessitate. It could be argued that there would be a difficulty in linking 3D sounds to 2D graphics in order to localise the sounds, and as such, a key focus of the iterative evaluations was the extent to which 3D sounds could be localised within the 2D visual space.

Game play begins with an introduction to the story behind Dartanan, and then players are given the option of playing the main platform game, or of calibrating the VHA within a “3DTI” section of the game in order to play the mini-games. In future, Children with hearing loss and their audiologist can then calibrate the VHA through this 3DTI section by listening to a sound source whilst adjusting the parameters until they hear the sound source well, and then revisit the main game to experience the sound using their new calibrations.

**Figure 2.**
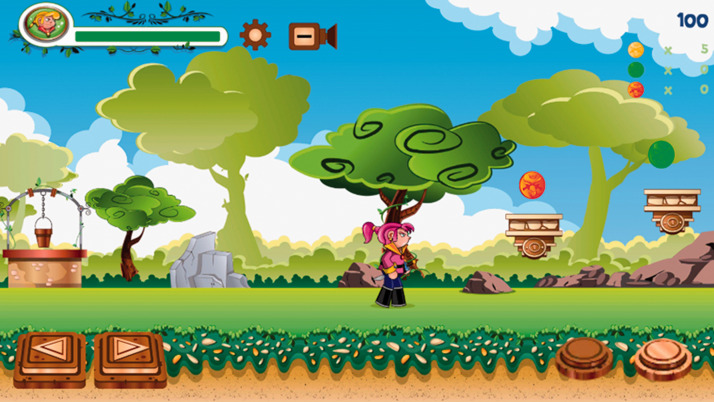
Screenshot of Dartanan’s main platform style game.

The main game (see Figure 2) has a character who jumps over platforms, fights with enemies, avoid traps, and collects coins following a quest to retrieve a stolen flower.

**Figure 3.**
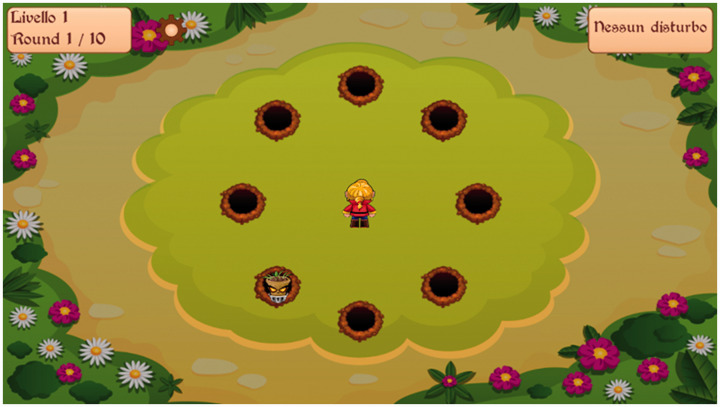
Screenshot of one of Dartanan’s mini-games designed for HA users.

The mini games (see Figure 3) are played in levels of increasing sound complexity. For example, there is a ‘whack-a-mole’ style game where players identify where an enemy is coming from by the direction of the sound. The levels become more varied by changing background noises. In this way, the child would be able to learn how to adjust microphone and noise reduction features of their HA to complete certain tasks (in this example, identify the direction that a sound is coming from).

**Figure 4.**
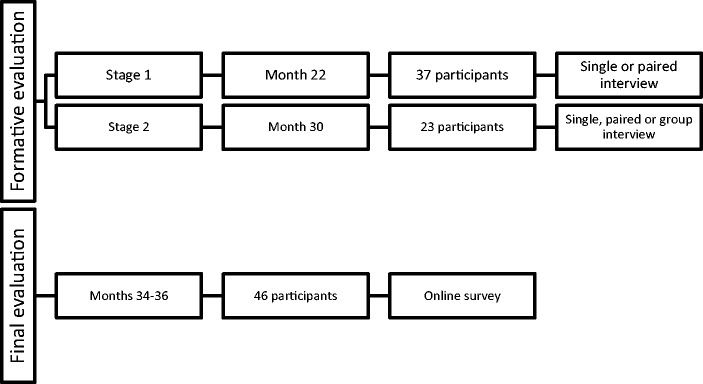
Stages of evaluation of Dartanan.

Dartanan is also intended to facilitate discussions of any issues children may have in completing this task with their audiologist as the audiologist is provided with data from the game-play, and children can discuss a recent, concrete experience.

### Study design

The research aim guiding the study was to perform user-evaluations of game prototypes to provide input for the further development of the game and to provide a final evaluation of the extent to which end-users thought that the game met its stated aims; empowerment and education for children with HAs; and engagement and usability for children with and without HAs. Children with and without HAs took part in two phases of studies in which they first evaluated prototypes (formative evaluations) and then evaluated the final version of Dartanan. Figure 4 details the stages of all evaluation activities.

### Research ethics

Ethics approval for the study was granted by the Faculty of Engineering at The University of Nottingham and at The University of Malaga. Regarding the part of the study carried out in Italy, in line with usual practices, no formal process was required and ethics approval was gained internally through the 3DTI Ethics Coordinator. Researchers in Italy followed research protocols designed by UK partners.

## Formative evaluations

A user-centered approach using interviews and questionnaires was employed for the formative evaluation stages. Participants were given the game prototype to play and were then asked a structured list of questions in either a single, paired or group interview. The purpose of the studies was to elicit subjective responses to the game, identify any potential problems as well as generate possible solutions for any problems that were identified. A bespoke approach to data collection (rather than the use of specific usability instruments) was utilised throughout the project for a number of reasons. Firstly, the main goal of the evaluations was to receive feedback on the extent to which the game met the project aims, which necessitated a bespoke interview/questionnaire schedule. Another important factor that influenced the design was its adaptability of the instruments to the differing situations in which data would be collected, for example within primary schools, or within audiology appointments. It was important to ensure that the questions were precisely targeted towards the project aims. Finally it was important that the data collection process could be used by multiple members of the project team in different countries in different languages, which necessitated a simplified approach.

### Participants

Children with and without HAs in the UK, Spain and Italy were invited to participate. Recruitment of participants in the UK was carried out by the University of Nottingham and Imperial College London. After consultations with audiology professionals, we selected a target population of children between the age of 8 and 18. We classified those under the age of 18 as children, as paediatric patients start to transition to being an adult patient between 16–18 with the actual transition happening at age 18. Whilst the game is targeted towards the younger end of this age range and there may be differences in game related preferences within the age range, the developmental age is also a factor. For example, an 18-year old with delay (resulting from hearing loss and/or other factors) may have a developmental age of 15. Thus recruiting children aged between 8 (considered old enough to engage in research activities) and 18 was thought to be inclusive.

Participants were contacted through emailing existing and personal contacts, adverts on social media, research websites and posters placed in audiology clinics in the UK. Recruitment and organisation of interviews with children in Spain was carried out by the University of Malaga and was assisted by a primary school and an audiologist who contacted the parents of clients who fit the criteria by phone. Recruitment, organisation and interviews in Italy were carried out by GN Resound assisted by the University of Padova and a hearing association. Audiologists known to the research teams were asked to contact parents of children with HAs by phone or email.

The recruitment criteria for end-users (as defined through consultation with hearing associations in the UK) was for children aged 8–18 years with mild to severe hearing loss who were users of behind the ear, in the ear, in the canal or completely in canal HAs. In total, 60 children took part in two phases of prototype evaluations of Dartanan. Participants are detailed in Table 1.

**Table 1.** Demographic information regarding participants of the formative evaluations of Dartanan.

Phase of formative development	Mean age (yrs)	Gender		Country	HA users	Children without HAs	Totals
		Female	Male				
Phase 1	10.35 (SD 1.38)	51.3%	48.6%	Italy	6	0	37
				Spain	0	12	
				UK	0	19	
Phase 2	11.22 (SD 2.21)	60.9%	39.1%	Italy	0	0	23
				Spain	11	0	
				UK	2	10	
Total					19	41	60

HA: hearing aid.

### Procedure

The procedure for the prototype evaluation was identical for both phase 1 and 2 although interview questions were different. Participants and their parents/guardians were invited to the research site. They were asked to first complete a short demographics questionnaire. Participants were given a short introduction to the game and were asked to play the game for approximately 30 minutes. Game play was followed by a single, paired or group (up to three participants) interview depending on the availability of participants. In phase 1, participants were asked about audiological aspects, game play and game story, game mechanics, accessibility, usability and aesthetic issues. During phase 2, participants discussed the extent to which they learned about HAs/hearing loss; the usability, acceptability, and audiological elements of the game; and the game’s relevance to the aims of the project. They were also asked about possible improvements to the game and about the mini-games specifically. All interviews were audio recorded.

The survey, interview protocols, information and consent forms were originally developed in English and were translated into Spanish and Italian. All of the interview recordings were transcribed verbatim and translated into English for ease of analysis. Survey data were also translated into English for analysis.

### Feedback from formative evaluations

The feedback derived from the interviews are summarised in Table 2, although it should be noted that developers were given an expanded version of these summaries, presented as positive feedback, negative feedback and ‘actionable changes’ (i.e. suggestions for improvement). These actionable changes consisted of proposals of simple solutions to problems identified by the participants along with the participants’ own suggestions for improvements. The human factors researchers and an audiologist, who had also played the game, suggested improvements based on the negative comments of the participants. The feedback relating to each area of investigation is outlined below along with suggestions that developers were given for improvements. Developers were encouraged to consider making either these suggested changes or to generate their own solutions to the identified problems before the final evaluation period.

**Table 2.** Summary of feedback and design impacts from the formative user-evaluations.

Category of evaluation	Positive feedback	Negative feedback	Design impacts for the developers
Audiological aspects	Participants liked the sounds and found that they were relevant to HA users. Some thought the game tasks were relevant to tasks that people would carry out in their daily life. HA users thought that the game would encourage them to find out more about HA functionalities and they could identify the resulting difference from the adjustments they made. Children without HAs thought that the game would be inclusive.	Participants thought that the main game was not related to hearing, and some hearing tasks (mini-games) were not solely hearing related, because they employed visual cues as well as auditory cues. The 3D sounds were not fully functional, and there was no difference in difficulty as players progressed through the levels of the mini-games. HA users would not do anything differently with their HA after playing Dartanan, and some thought it would not be a useful addition to audiology appointments.	• Increase the relevance of the main game and mini-games to hearing loss and HAs by introducing a common theme throughout the games and providing more instructions about the VHA and mini-games.• Provide Dartanan with a hearing related motivation in the main story.• Visual cues should be reduced in the mini-games.• Ensure that the 3D sounds are functional on different kinds of device.
Learning about HAs	Some participants without hearing loss found that after using Dartanan they had learned a little about HAs and communication practices to help those with hearing difficulties. They also learned a little about what life is like for those with hearing loss. Participants liked the promise of being able to learn whilst playing a game.	The HA related aspects of the game were not adequately described, meaning participants did not know what they were supposed to learn. Many of the participants without hearing loss reported that they did not learn anything about HAs and those with hearing loss found that the game did not teach them much about HA settings.	• Characters and tasks should be more relevant to hearing/hearing loss.• Improve descriptions of the VHA and audiological terminology throughout.
Game play and game story	Participants with and without hearing loss enjoyed the platform aspect of the main game, they liked the increasing challenge, and they enjoyed the enemies and characters which made the game fun to play. Most understood what they had to do in the game, they thought that it gave good feedback and they liked how the game was inclusive.	Participants found some challenges in the main game too difficult; they did not understand the rewards and coins, they found that they did not always understand what to do in the mini-games, and they found the variety of enemies and tasks confusing.	• The mini-games need instructions that appear before players encounter the game.• Begin mini-games at an easier level and increase the difficulty as the participant progresses through them, or introduce an “easy” mode.• Make the solutions to crucial tasks more obvious.• Explain what the coins are to be used for in the introduction, and ensure the totals are accurate.
Game mechanics	Participants thought that the main game was easy and intuitive to control, and that the buttons made a satisfying click when pressed. Players were also satisfied with the feedback provided on how much ‘life’ their character had left.	Participants did not like the lack of checkpoints which made the main game too challenging. They thought the controls were not obvious and it was not obvious how to succeed in crucial parts of the level. Sometimes the character did not move as expected when the button was pressed.	• Include more checkpoints• Ensure that Dartanan has full health at the start of each new level.• Controls need to be responsive immediately. Consider adding a visual, haptic or sound cue to acknowledge a button press.
Accessibility		Participants did not note any difficulties based on their own abilities, but they thought that the controls might be too difficult for young children to use owing to their placement on the tablet device.	• Game controls should be made easier for younger children, or their placement should enable children with smaller hands to play on larger devices.
Usability and aesthetics	Participants enjoyed the graphics and thought that the character was gender neutral. They liked that there was not a tutorial that delayed their playing, and thought that the game would be suitable for different types of device. Those that found the instructions thought that they were useful.	The loading screen was thought to be too boring and that the controls were not always obvious in certain types of backgrounds making it difficult to find the crucial items needed to progress to the next level of the main game. Not all participants found the instructions, and some did not know how far they had progressed in the game.	• Ensure that the control buttons are more visible in all backgrounds.• Include more obvious instructions both as a separate instructions section and built into the game.• Include a map of the “world” and information about how their progress relates to Dartanan’s overall quest.• Make important features eye-catching and obvious.
Acceptability	Participants with and without hearing loss liked the graphics, music, adventure and the character. HA users thought that the concept was good, they liked being able to hear more clearly and they thought other HA users would like to use this game with their audiologist to learn about HAs.	Some participants with and without hearing loss found the music annoying, and did not like certain enemies and sounds, for example some of the monsters made an unpleasant noise. Some found there was not enough positive feedback about their progress to encourage them to keep playing.	• Give more feedback about achievements.• Change the sounds that enemies make.• Increase the relevance Use different music for each level.

HA: hearing aid; VHA: virtual hearing aid.

**Table 3.** Participants involved in the final evaluation of Dartanan.

	Project partner	HA users	Children without HAs	Gender			Total
				Male	Female	Prefer not to say	
Final evaluation	Italy (GN Resound)	9	0	3	6	2	46
	Italy (XTeam)	33	4	16	19	0	
Totals		42	4	19	25	2	

HA: hearing aid.

## Final evaluation

For the final evaluation stage during months 34–36 of the project, participants were given the final prototype to play at home or in an audiology clinic. A survey method was used to capture feedback on this prototype which was completed immediately following game-play. The purpose of this evaluation was to validate the extent to which the game met the project goals.

### Participants

For the final evaluation, recruitment was carried out by project partners GN Resound and XTeam, both in Italy. Recruitment criteria and approaches were the same as those used during the formative evaluations. During the final evaluation, children aged 8–18 took part in an online survey after playing the game for a minimum of 30 minutes. Table 3 details the demographics of these participants.

### Procedure

For the final evaluation, parents/guardians were asked to either bring their child to the research site, or they were sent a link to a website where they could download the game to play at home. Children were asked to play Dartanan for at least 30 minutes before visiting an online survey to answer questions about the game. The survey focused on the extent to which participants thought the game met the project aims for the creation of an inclusive game for all children, which also teaches children with HA about the settings and features of their HA in order for them to feel comfortable discussing these with their audiologist.

The survey began with some open questions regarding what participants liked, disliked and things that they thought should be changed about Dartanan. All participants answered Likert style questions (strongly disagree, disagree, neither agree nor disagree, agree or strongly agree) which concerned the acceptability of the pricing of the app (a price was given in the local currency) and whether or not they would buy the app (or ask someone to buy it for them); whether they would recommend it to their friends; and whether they had any further comments about Dartanan. Children with HAs were also asked whether Dartanan helped them to learn about what the different settings/features of their HA do; whether they learned about when to use the different settings/features of their HA; whether they were more likely to use their HA after using Dartanan; whether Dartanan would encourage them to speak to their audiologist about HA settings/features; whether they feel more confident using technical words relating to their hearing after playing Dartanan; and whether it would be useful for them to play Dartanan. All of these questions were presented along with an optional comment box, which invited participants to explain their responses.

### Final evaluation findings

As is reasonable to expect from child participants, the open questions regarding what participants liked and disliked about the game were met with short and simple responses. The features that participants liked were the graphics, the character Dartanan, the movements of the character (jumping and fighting), and the number of levels to play. The game was described as ‘fun’, ‘funny’ or ‘amusing’. One participant said that they found it easy to learn, and liked that there were many levels to play. One person said it was a ‘beautiful game’.

Conversely, some participants disliked that there were ‘too few levels’, that the game was ‘too short’ and some technical issues were commented on such as ‘sometimes it doesn't work on tablet’ or that the game was slow. One person commented that the game was ‘too male design’ and one person disliked that ‘there is no final ranking with scores’.

Suggestions for improvement were similarly restricted. Comments include ‘more levels’, ‘more levels-characters and enemies’, ‘more female design’, ‘fix the bugs’ and ‘more instruction for mini-games’.

**Figure 5.**
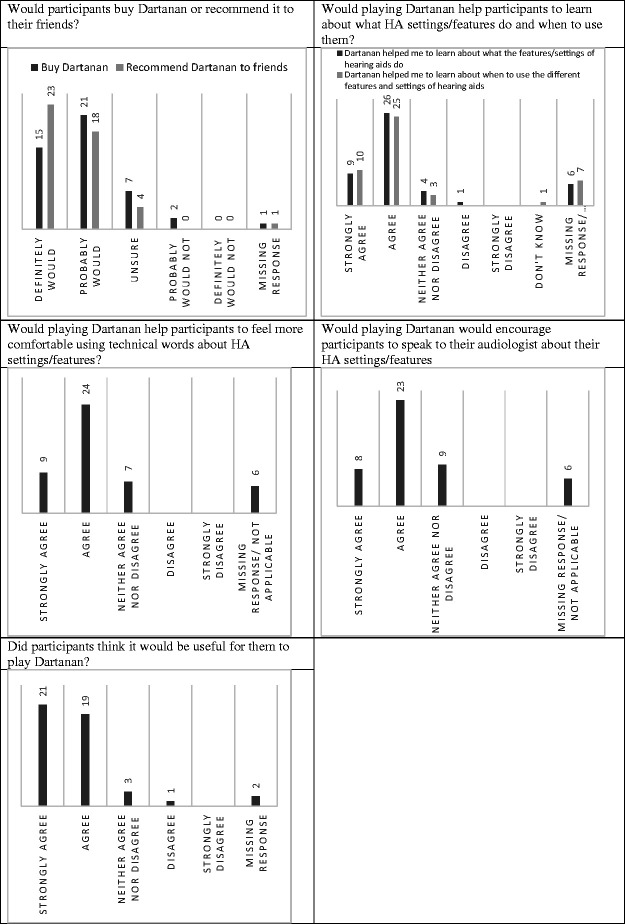
Participants responses to Likert style questions regarding the apps stated aims.

Participants’ responses to Likert style questions relating to whether or not the game met its stated goals are presented in Figure 5.

The majority of participants stated that they would probably buy the game whilst the rest were unsure. The majority also stated that they would probably or definitely recommend the game to others. Regarding the app’s aims, the majority of participants either agreed or strongly agreed that Dartanan helped them to learn about what different HA settings do, or when to use the different settings. Of the minority of participants who selected “don’t know”, one participant pointed out that their HA settings were automatic so they would have very little opportunity to examine the settings after playing the game.

Most users would feel more comfortable in using technical words (such as compression and directionality) after playing Dartanan. No participants gave reasons for their responses to this question. Participants were asked whether using Dartanan would encourage them to speak to their audiologist about their HA settings/features. Most agreed that it would. No participants gave reasons for their responses to this question. Finally, participants were asked whether it would be useful for them to play Dartanan. Most participants selected either ‘agree’ or ‘strongly agree’.

Overall, participants thought that the game met its stated goals. Children thought it would be useful and informative to play Dartanan. Very few areas of improvement were identified for the developers to act upon.

## Discussion

Dartanan aims to teach children with HAs about the functions of their HA as well as being a fun game for all children to play. The studies outlined in this paper describe game prototype evaluations with 106 children with and without HAs. Two formative evaluation stages required children to play the game for 30 minutes and give their feedback within paired, single or group interviews. Children also participated in a final evaluation in which they were asked to give their feedback using an online survey. Findings show that the game concept is acceptable to children with and without hearing impairments. Participants were positive about the game concept and thought that it was inclusive. Throughout the formative evaluations, it was clear that children thought that the main game was fun and engaging and participants found the visuals attractive. Some participants without hearing loss learned a little about hearing loss and all learned a little about HA functions. However, the game had not yet met the stated project goals as evidenced by the negative aspects of the user-evaluations relating to learning and audiological aspects.

The developers were given detailed feedback based on the user-evaluations, and encouraged to make these changes for the final evaluations. During the final evaluation, participants thought that the game met its stated goals. Specifically, Dartanan helped them to learn about what different HA settings/features do, and also about when to use the different settings/features. After using Dartanan, participants would feel more confident using technical terms relating to HA settings/features. Most thought it would be useful for them to play Dartanan. Participants with HAs also felt encouraged to speak to their audiologist about HA settings/features. This suggests that Dartanan might prove to be an empowering tool for children which might help them to speak up for their own needs and preferences. Moreover, it seems that the user feedback that was provided to the developers helped to guide the development process towards a useful product.

The engagement of two user groups (children with and without HAs) in these evaluations enabled us to gain an understanding of the requirements of this dual-purpose game. Through the iterative design and evaluation process, the developers were provided with many suggestions both from their end-users and the researchers about ways in which the game could be improved in order to increase the opportunities for learning and engagement. The paper reinforces the importance of accessing and understanding user’s experience in order to help developers to focus on users’ needs. That the game was rated positively in the final evaluation is reflective of the value of the evaluation process to the end-product. However, it is acknowledged that since the measurement tools rely on self-response, there may be bias in the ratings and reactions of the users, which limits the extent of conclusions that can be made here in relation to efficacy of the game in HA success. For this reason, the next stages of research in this area should focus on more objective measures, such as audiologists measures of improvement in hearing outcomes after children’s engagement with the game.

### The promise of Dartanan for audiological services

The finding that children with HAs found the game engaging is valuable in many ways. Children with hearing impairments might be encouraged to use the game regularly, and therefore increase their knowledge and experience with HA settings in order to become familiar with their hearing loss and what their HAs could do for them. This might also increase the amount of time that children use their HAs and potentially increase their adherence to the use of HA as they enter crucial formative years. Indeed Walker, McCreery et al.^36^ suggest that more regular training interventions might increase the consistent usage of HAs in children, specifically providing hearing loss simulations and providing examples of listening in noise with and without HAs. These kinds of hearing tasks were presented in the mini-games, so it is possible that games like Dartanan could provide an engaging means of training using listening in noise with and without the user’s HA or the game’s VHA.

Building a feeling of empowerment within children with HAs is important.^20^ Overall, children with HA who evaluated the final app thought it would be useful to play Dartanan. They also thought that using Dartanan would encourage them to speak to their audiologist about their HA settings. This is an important finding in the context of the likelihood of non-use of prescribed HAs amongst children.^36,37^ Given that participants thought that Dartanan would help them learn more about what their HA does, it is possible that children would become more informed about what benefits their HA could bring. Furthermore, if Dartanan could help children to feel more empowered to learn about and discuss changes to their HA, it is possible that they might feel encouraged to seek help and assistance in other matters relating to their hearing, and to take control of their HAs.

In a study by Elkayam and English^38^ hard of hearing adolescents valued learning new information about their hearing loss as a result of completing self-assessment questionnaires such as the SAC-A/SOAC-A questionnaires.^10^ The authors refer to the self-assessment practice as a ‘*nonintrusive*’ means of stimulating conversation (p.495).^38^ However this practice implies that adolescents have sufficient knowledge and understanding of their own hearing loss and how it impacts on their daily life to be able to accurately complete the self-assessment materials. It is possible that Dartanan’s value lies in the affordances for children with hearing loss to examine the extent of their hearing loss in order to discuss it with relevant caregivers. Dartanan could be used in conjunction with other self-assessment to monitor both affective and functional abilities. The data created during their engagement with the game would provide objective feedback (i.e. their performance in the games) about their hearing ability to complement their own and their parents’ subjective feedback. From the point of view of the audiologist, the data may inform important choices made during programming. For example, if the child had a tendency to use a split directional microphone array in favour of an omnidirectional or fully directional setting, this would provide useful insight to the child’s personal preference regarding microphone arrays set-up. This type of preference would be difficult for the child to communicate to the audiologist or to absorb from the parental feedback.

### Conclusions and next steps

Dartanan was thought to be an engaging and fun game to play for all children. In the first two evaluation phases, it was found that the aspects of the game directed at hearing loss and education about HA functionality were not fully meeting the objectives of the 3DTI project. The developers were provided with suggestions for making improvements that would improve the game towards the objectives of the project. After redevelopment based on these changes, the game was judged to have met the project goals by most participants during the final evaluation. Participants in the final evaluation thought they would benefit from Dartanan in terms of learning about the settings/functions of HAs and thought it would be a useful addition to their audiology service. Increased knowledge of the HA function may boost the child’s HA self-efficacy, which has been shown to be an important factor in the adult HA user population for HA use and engagement in their rehabilitation [35]. It is important to engage children in their HA care, at an early stage, as their hearing loss is likely to be a long-term life condition that will need managing with hearing devices. It can also be concluded that the project’s goal of empowering children to feel more in control of their disability has also been addressed by the game.

Comparison of the findings of the prototype evaluation with the findings of the final evaluation suggests that the process had a positive effect on the educational potential of the game. Providing that the small number of issues identified in the final evaluation are addressed by the developers, it seems that overall Dartanan should be well received by its target audience. Moreover, it is possible that Dartanan will improve the experiences of children with HAs by improving their knowledge of their HA and empowering them to take an active role in their hearing impairment and HA.

The natural next stages of research are to evaluate games like Dartanan with larger populations and using extended periods of game play. It might also be useful to carry out tests on the extent to which such games can improve the HA fitting process for children and audiologists. More objective measures would be required to assess the efficacy of the game in improving HA outcomes for children and therefore increasing usage and uptake of HAs amongst children who need them.

## Notes

I
https://itunes.apple.com/gb/app/hearing-loss-simulator/id398352094?mt=8

II
http://www.starkey.co.uk/hearing-loss-simulator

III
https://www.phonak.com/uk/en/hearing-loss/signs-of-hearing-loss-and-what-to-do/hearing-loss-simulation.html

IV
http://www.ndcs.org.uk/family_support/childhood_deafness/what_does_a_hearing_loss_sound_like/index.html

V
https://www.hearinglikeme.com/hearing-loss-simulator/
